# A Systematic Review and Narrative Synthesis of Cognitive Training in the Treatment of Mental Illness and Substance Use Disorder

**DOI:** 10.3390/jcm13154348

**Published:** 2024-07-25

**Authors:** Kerri M. Gillespie, Alexander H. Dymond, Xin Li, Daniel Schweitzer, Grace Branjerdporn, Saleha Khan, Quang Hii, Suzie Keller, Selena E. Bartlett

**Affiliations:** 1 School of Clinical Sciences, Translational Research Institute, Faculty of Health, Queensland University of Technology, Kelvin Grove, QLD 4059, Australia; selena.bartlett@qut.edu.au; 2 Gold Coast Hospital and Health Service, Southport, QLD 4215, Australia; alex.dymond@health.qld.gov.au (A.H.D.); saleha.khan@health.qld.gov.au (S.K.); 3 Mater Hospital, South Brisbane, QLD 4101, Australia; xin.li2@griffithuni.edu.au (X.L.); daniel.schweitzer2@mater.org.au (D.S.); grace.branjerdporn@mater.org.au (G.B.); 4 Forensic and Secure Services, The Park—Centre for Mental Health, Wacol, QLD 4076, Australia; qk.hii@health.qld.gov.au (Q.H.); suzie.keller@health.qld.gov.au (S.K.)

**Keywords:** cognitive training, cognitive remediation therapy, CRT, cognition, mental illness, executive function

## Abstract

**Introduction:** The one unifying and distinguishing feature of all neuropsychiatric illnesses is the co-occurrence of cognitive dysfunction. Cognitive training (CT) was developed to enhance neural connectivity and cognition and improve day-to-day functioning. However, the benefits of CT are still debated. This current systematic review aimed to examine the efficacy of CT and to identify diagnostic and CT characteristics associated with superior outcomes across a range of psychiatric disorders. **Method:** Studies investigating CT in psychiatric illnesses were extracted from Embase, PubMed, CINAHL, PsycINFO, and PsycARTICLES up to 17 August 2023. Inclusion criteria were randomised control trials (RCT) and English language. The primary search strategy included terms relating to cognitive training, cognitive remediation, cognitive enhancement, or cognitive rehabilitation and randomised control trials, clinical trials, or experiments. Risk of bias was assessed using RevMan Web version 8.1.1. Narrative synthesis was used to analyse findings. Due to the heterogeneity of participant demographics, diagnoses, and interventions, meta-analyses were considered inappropriate. **Results:** Fifteen studies, including a total of 1075 participants, were identified. Approximately 67% of studies reported significant improvements in at least one trained domain of cognitive function after CT, and 47% observed improvements in psychiatric symptoms or function. Cognitive transfer effects were not observed. Sample sizes for studies were generally small, and most CT durations were 6 weeks or less. **Conclusions:** Findings suggest that CT can improve cognitive function in trained domains, though little evidence of cognitive transfer effects was observed. Due to the lack of standardisation in CT format and delivery, and inadequate measures of psychiatric symptoms or daily function, there is insufficient evidence to conclude whether or not this technique may benefit cognitive impairment in psychiatric disorders, or lead to subsequent improvement in disease symptomatology. Further studies of longer duration and using consistent methodologies must be conducted to identify the benefits of CT in psychiatric disorders.

## 1. Introduction

Cognitive dysfunction is one of the most commonly reported and consistent impairments in psychiatric disorders across the lifespan [1]. A treatment technique that has been proposed to reduce, or possibly ameliorate, the cognitive and functional impairments that may occur across the spectrum of psychiatric disorders is cognitive training (CT). CT uses the systematic repetition (drill and practice) of tasks designed to target one or more cognitive functions, traditionally memory, attention, cognitive flexibility, processing speed, and social cognition [2]. Some studies incorporate strategy training, which evidence suggests may elevate the results [3]. CT is also known as cognitive rehabilitation, cognitive enhancement, or cognitive remediation therapy (CRT). While these terms are often used interchangeably in the literature, CRT is a specific form of CT that must also include the core components of a trained therapist facilitator, the development of cognitive strategies, and procedures that support the real-world transfer of skills [4].

Psychiatric disorders with comorbid cognitive impairments are often characterised by reduced cortical volume and abnormal brain networks [5,6]. CT is grounded in the principle of neuroplasticity which hypothesises that repeated cognitive engagement will lead to beneficial changes in cortical density and organisation [7]. According to this theory, cognitive engagement can lead to adaptive changes in the cortical density and organisation in the brain, which may improve across the different cognitive domains and subsequent improvements in symptoms and daily function. Previous neuroimaging studies in older adults investigating the clinical application of CT have shown adaptive neuroplastic changes and increased functional connectivity following a training period [8,9]. A meta-analysis showed that CT was associated with increases in activation in the left inferior frontal gyrus, an area associated with language, working memory, cognitive control, and imagination [10].

A large amount of overlap is seen in the cognitive deficits and training techniques used in different psychiatric disorders. A broad range of cognitive deficits have been observed in mood disorders (depression and bipolar affective disorder), most commonly in working memory, memory, processing speed, inhibition, attention, and executive function [11]. Rumination, thought to directly burden and impair working memory [12], is also characteristic of depression and bipolar disorder [13]. Working memory training is frequently used as a technique to diminish rumination and reduce depressive symptoms. However, past studies have shown inconsistent results [14,15].

Attention bias toward negative stimuli is another common feature of depression that may contribute to the development, maintenance, and severity of the disorder [16]. This trait is also seen in individuals with substance use disorder, who commonly show selective bias toward cues related to substances, further driving cravings and substance use behaviours [17]. Attention bias modification techniques are widely used in both depression and substance use disorder (SUD), though there is no consensus on the benefits of this treatment method [18,19]. SUD is also associated with impairments of working memory, inhibition, and delay discounting [2]. Working memory is strongly associated with executive function, decision making, and self-control, all of which contribute to poor outcomes in treatment, indicating working memory may be an ideal CT target for individuals with substance use disorder [20,21].

Eating disorders (ED) and obsessive–compulsive disorder (OCD) have long been associated with one another, due to a number of shared traits such as weak central coherence, cognitive inflexibility, and attentional bias [22,23,24]. Weak central coherence causes individuals to fixate on small details, experiencing difficulty integrating details into a whole [25]. Previous studies have used visual organisational strategies to improve these deficits in OCD, which have seen improvements in memory and clinical symptoms [26,27]. These strategies may also see improvements in anorexia nervosa (AN), although more comprehensive CRT treatment modalities are more commonly used in eating disorders [25]. Cognitive bias modification may also provide a beneficial tool for managing individuals with OCD and ED.

Although extensive studies have been undertaken in CT for psychiatric disorders, there remains considerable inconsistency in results and variability in targeted domains, training procedures, assessment methods, and follow-up durations, which means that the comparison and evaluation of CT have been difficult to assess in clinical practice [2]. Whether training has transfer effects to non-trained cognitive domains or daily function is also contested [28,29,30]. Previous studies have included a wide range of alternative CT treatments or non-CT active treatments as control groups. To obtain a less confounded insight into the impact of CT on cognitive and clinical outcomes for those with psychiatric disorders, this review only includes interventions with non-active (treatment as usual or no care) control groups. This review also excludes schizophrenia-spectrum disorders, as several systematic reviews of CT and schizophrenia have been undertaken. These found improvements in targeted and non-targeted cognitive domains and daily function [31,32,33,34]. Previous systematic reviews have addressed CT in a singular diagnostic population [25,35], or via one administration method [36] which impedes investigation into the differential efficacy or specific effects across the diagnostic and implementation groups. Hence, this systematic review has gathered a wide range of studies investigating the impact of CT on cognitive deficits associated with psychiatric disorders other than schizophrenia to develop a more nuanced understanding of the potentially beneficial aspects of CT and its role across a broad range of mental health clinic settings.

## 2. Aims

### 2.1. Primary Aims

1. To evaluate the impact of CT on cognitive outcomes in people with a psychiatric disorder other than schizophrenia.

### 2.2. Secondary Aims

1. To evaluate the impact of CT on clinical outcomes (e.g., psychosocial function, depression, or substance use) in people with a psychiatric disorder other than schizophrenia.
2. To determine the differential impacts of CT across psychiatric diagnoses.
3. To identify whether specific CT characteristics or formats provide superior outcomes.

## 3. Materials and Methods

This systematic review was conducted in accordance with the Preferred Reporting Items for Systematic Review and Meta-Analyses (PRISMA) guidelines [37]. A narrative synthesis of studies was conducted. The risk of bias analysis was conducted using the Cochrane Review Manager Web (RevMan Web) version 8.1.1 (the Cochrane Collaboration, Copenhagen, Denmark). This study was registered in the International Prospective Register of Systematic Reviews (PROSPERO; ID: CRD42023461666).

### 3.1. Eligibility Criteria

#### 3.1.1. Participants

This review has assessed all eligible sources containing participants who are identified as having a psychiatric disorder other than schizophrenia or psychosis, as defined by the International Classification of Diseases 10th Revision (ICD-10) criteria. Studies were excluded if they included participants who were identified as being healthy, having a schizophrenia-spectrum or psychotic disorder, having age-related cognitive decline (including mild cognitive impairment and dementia), having a traumatic brain injury or cognitive impairment caused by physical injury, or cognitive impairment due to chromosomal abnormalities or inherited genetic conditions. We have also excluded studies that examined psychiatric disorders that are considered to have a developmental basis (e.g., autism spectrum disorder and attention deficit/hyperactivity disorder (ADHD).

#### 3.1.2. Interventions

This study investigated the use of CT to improve or enhance cognitive function. This may also be known as cognitive rehabilitation, cognitive remediation, or cognitive enhancement therapy. Any studies that include a therapy model that does not target one or more domains of cognitive function, or provide a measure of cognitive function as an outcome, were excluded (including cognitive behavioural therapy or mindfulness therapies). Interventions may be administered by various mental health clinicians, including psychiatrists, clinical psychologists, occupational therapists, mental health nurses, or social workers.

#### 3.1.3. Study Type

This review limited all searches to randomised control trials utilizing primary data. This limitation increased the reliability of the findings. Studies must include a treatment-as-usual or no-treatment control group. Studies that include an active control group were excluded from the review. All reviews, opinion and commentary papers, posters, and conference proceedings were excluded from the study.

#### 3.1.4. Outcomes

The study’s primary outcomes are a change in one or multiple domains of cognitive function (such as memory, flexibility, planning, attention, or executive function). Clinical outcomes (such as daily function, depression, anxiety, or quality of life) were also extracted and analysed where possible. Moderating factors, such as CT dose and frequency, disease severity, CT type, and delivery method were investigated where possible for their impacts on findings.

### 3.2. Search Strategy

The databases searched were Embase, CINAHL (via EBSCOhost), PubMed, APA PsycINFO (via EBSCOhost), and APA PsycARTICLES (via EBSCOhost). The primary search strategy was (“cognitive train*” OR “cognitive remediation” OR “cognitive rehabilitation” OR “Cognitive therapy” OR “Cognitive enhancement” OR “brain train*”) NOT (“intellectual disability” OR “mental retardation” OR “brain injury” OR “stroke”) AND (random* OR “randomized control*” OR “randomised control*” OR trial OR “clinical trial” OR “clinical study” OR control* OR crossover OR “cross*over” OR parallel OR compar* OR experiment*). The reference lists of all included papers were screened for additional papers. The reference lists of other completed systematic reviews on similar topics were also searched to ensure no papers were overlooked. No date restrictions were placed on the search. However, only papers available in English were included.

### 3.3. Study Selection

Following searches, all identified papers were imported into COVIDENCE for screening, and duplicates were removed. The screening procedure was carried out in two stages. Two researchers independently screened papers by title and abstract for inclusion in the first stage. The second stage comprised a full-text screen conducted by two independent reviewers. Any disagreements were resolved by a third reviewer, who investigated papers under dispute.

### 3.4. Quality Assessment

Two reviewers independently assessed each included paper for risk of bias using RevMan Web (the Cochrane Collaboration, Copenhagen, Denmark). This tool measures bias in the individual domains of selection bias, detection bias, performance bias, attrition bias, and reporting bias.

### 3.5. Data Extraction

Data were extracted based on strict criteria (population demographics and diagnosis, study design, intervention and placebo type, and outcome) which were recorded using a predefined working spreadsheet. Where data are missing from an evidence source, authors of the articles were contacted with a request for these missing data. Data extracted from the identified papers included general characteristics of the study (year, location, sample size, follow-up, and duration); characteristics of participants (age, diagnosis, ethnicity); setting (inpatient, residential, or community); characteristics of the intervention (cognitive domain targeted); and outcomes (results, side-effects and complications, follow-up duration, and attrition).

## 4. Results

### 4.1. Study Selection

**Figure 1:**
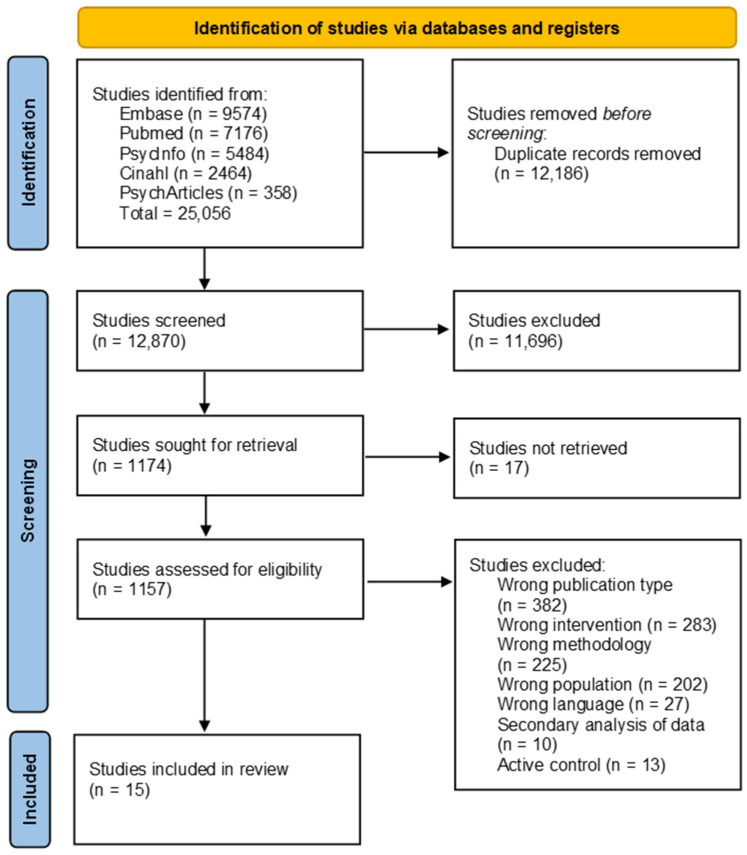
PRISMA study flow diagram [37].

The search strategy is depicted in a PRISMA flow diagram in Figure 1. The initial search resulted in 25,056 published studies. After excluding duplicates, 12,870 remained for title and abstract screening. Seventeen of these papers could not be located. Full text screening of the remaining 1157 articles excluded a further 1142 articles for being abstracts, reviews, posters, or another ineligible publication type; using the wrong participant population, methodology, intervention, or language; or for containing a secondary analysis of previously published data. A hand search was also conducted of references lists of related systematic reviews and manuscripts, but no further articles were identified.

### 4.2. Study Characteristics

Our systematic review identified and analysed 15 articles that studied CT in participants with depression (n = 6); substance use disorder (n = 3); bipolar affective disorder (n = 3); anorexia nervosa (n = 2); and OCD (n = 1). In total, 1075 participants were enrolled in all studies, including 573 inpatients and residential patients, 362 outpatients, and 140 community members. Thirteen studies included measures of psychosocial function, quality of life, or disease symptoms, such as depression, anxiety, eating disorder severity, craving, self-control, and risk-taking. See Table 1 for full study characteristics.

### 4.3. Risk of Bias

**Figure 2:**
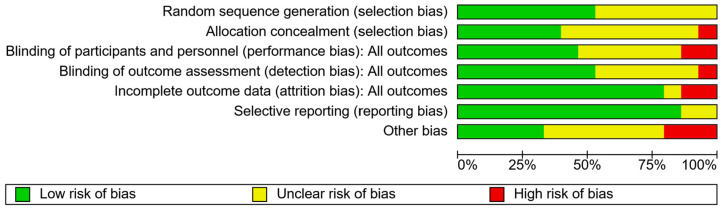
Risk of bias graph.

Overall (Figure 2) it can be observed that the main biases were incomplete outcome data (attrition bias) and blinding (performance bias). Participant attrition was a common aspect of the included studies, which posed an even greater risk due to the already small sample size of most studies. Blinding of researchers and assessors was commonly not mentioned or could not be carried out due to the nature of the study. ‘Other bias’ reflected the failure to include sample size calculations, underpowered sample sizes, or the failure to use intention to treat analysis after participant drop-out. Low risk of bias was seen most commonly in selective reporting (reporting bias). See Supplementary Figure S1 for an overview of risk associated with each paper.

### 4.4. CT Characteristics

Seven identified studies included six different commercially available, computerised training programs for general cognitive function (n = 4) [38,40,41,43,47,48], addiction (n = 1) [44], or schizophrenia (n = 1) [48]. All of these were therapist-led, and two included psychoeducation. One study used an adaptation of an existing block design task as their cognitive training [27]. Two studies developed their own cognitive training programs: a virtual reality platform (the Systemic Lisbon Battery) [45], and an addiction-specific mobile app [46]. The five remaining studies used existing, or researcher designed, manualized programs for in-person and group training [39,42,49,50,51]. Eight studies adhered to the CRT criteria outlined by Bowie, incorporating encouragement, strategy coaching, and transfer to real-life demands [27,38,41,47,48,49,50,51,52,53]. Two articles claimed to incorporate CRT [40,43] but did not appear to fit the criteria according to Bowie and colleagues [4]; therapists or experimenters were present for the training, but had minimal input. All studies used drill and practice approaches to training, while ten additionally included some form of strategy coaching (see Table 1).

### 4.5. Cognitive Domains

Five studies used domain-specific training, targeting working memory [44], memory [39], autobiographical memory [42], cognitive flexibility [51], or visual organisation strategies [27]. Autobiographical memory training for five weeks found significant improvements in memory and depression symptoms. Training in visuospatial organisation strategies over five weeks saw improvements in visuospatial construction, memory, and OCD symptoms. The other, three-to-five-week training strategies found no improvement in cognition, mood, or function. All other CT interventions targeted multiple domains of cognition. Of these, eleven targeted memory or working memory, nine targeted attention, and eight targeted executive function or cognitive flexibility. A minority of studies also included visual organisation strategies [27], visuomotor skills [43], processing speed [38], and inhibition [40,41]. Only one study incorporated attentional bias modification (included together with working memory training) [46]. This was conducted in inpatients being treated for methamphetamine use disorder using independent training on a mobile addiction app. This study found improvements in working memory, verbal learning, and decision making, but no improvements in attention bias.

### 4.6. Cognitive Outcomes

Only one included study used independent computerised training [46], which found improvements in trained cognitive domains of working memory as well as verbal learning in individuals with methamphetamine substance use. Five of the seven computer-based training interventions that were therapist-led [38,40,43,45,48], and both computer-assisted training interventions that included psychoeducation [41,47], led to improvements in at least one of the trained cognitive domains. One of the computer-based training interventions utilised virtual reality (VR) to create real life scenarios [45] leading to improvements in the trained domains of attention and cognitive flexibility.

Five articles described interventions that did not include computerised elements (both AN studies [50,51], one bipolar study [49], an OCD study [27], and one study of depression [42]). While two identified improvements in memory [27,42], these studies were less likely to find improvements in cognition than computerised tasks. The 21-week intervention conducted by Torrent and colleagues [49], focusing on function as well as cognition, found no improvements in any measure of cognitive function. The two manualized CRT interventions for AN [50,51] failed to identify any improvements in cognition. The four studies that failed to identify improvements in any measure of cognition [39,44,50,51] were all conducted at inpatient facilities.

### 4.7. Clinical Outcomes

Thirteen studies included a measure of clinical outcomes, including five that measured psychosocial function, and nine that measured psychiatric symptoms and related factors, such as depression, anxiety, OCD symptoms, Eating Disorder Examination Questionnaire (EDEQ) severity, risk-taking, self-control, and craving. Two studies conducted in SUD measured outcomes associated with addiction (risk-taking, self-control, or craving). Brooks and colleagues’ [44] study of working memory training led to no improvements in craving and self-control. The mobile addiction app, training working memory and attentional bias, saw improvements in risk taking [46]. Eight studies measured depressive symptoms (five in depression, and one each in AN, SUD, and OCD). Of these, only two studies in individuals with depression saw improvements in this symptom, alongside an improvement in attention [40] or memory [42]. Two studies that reported improvements in attention [41] or memory [27,43] found no associated improvements in depressive symptoms. Of the six studies that found no improvement in affective symptoms, four were conducted in inpatient facilities [39,43,44,50].

Psychosocial functioning was measured in all three studies of individuals with bipolar disorder, and in two studies of depression [38,41]. Only two therapist-led, computer-assisted studies for depression [41] and bipolar disorder [48] saw improvements in this measure. Torrent and colleagues [49] reported improvements in functioning compared to treatment as usual, but these improvements were not significantly better than the psychoeducation group. Of the two psychotherapy-based cognitive flexibility training interventions for inpatients experiencing AN, only Dingemans and colleagues [50] measured affective outcomes or eating disorder symptoms. Depression and anxiety were not significantly improved, but eating disorder severity and quality of life were improved at six weeks and six months, respectively. The sole study in outpatient individuals with OCD, using a therapist-led block design intervention, measured depression, anxiety, and OCD symptoms [27]. Improvements were observed in OCD symptoms only.

### 4.8. Duration

Studies were conducted for between 3 and 36 sessions over the course of 3 to 21 weeks. There was no obvious relationship between intervention length and cognitive or functional improvement. Computer-assisted psychotherapy interventions and pen-and-paper based CRT interventions tended to be slightly longer, with an average duration of 8.5 and 8.4 weeks, respectively. However, these more psychotherapy-driven models saw fewer improvements in cognition than did purely computerised training that targeted multiple cognitive domains. Participants had between one and five CT sessions per week. All studies tested participants pre- and immediately post-intervention. Four studies included follow-up measures after an additional 12 weeks [47,48], two months [42], and six months [50]. All four studies found additional improvements at follow-up that had not been observed at post-intervention, while other improvements (seen in function and disease severity) were no longer evident.

## 5. Discussion

This systematic review aimed to evaluate the efficacy of CT for improving cognition, symptomatology, or daily functioning in psychiatric illnesses other than schizophrenia. A comprehensive literature search identified 15 RCT studies without active control groups, and the types of CT were explored. Narrative synthesis of the included articles found little consistency in terms of CT format, targets, delivery, or of outcomes measured. Three main approaches to CT were identified: computerised training with little or no therapist involvement; computer-assisted training with significant therapist involvement and additional strategy coaching or psychotherapy; and non-computerised training relying solely on therapist training. Computerised interventions appeared to be superior to non-computer-assisted intervention in terms of cognitive improvements. However, a number of moderating factors may have influenced these findings, such as diagnosis and disease severity, treatment frequency and dose, treatment objectives, and degree of therapist involvement.

There was no evidence for the effectiveness of working memory training alone. Studies that found no improvements in any measure tended to be domain-specific and also shorter in duration (three to five weeks in length). However, no clear relationship was found between treatment dose or frequency and outcomes reported. In fact, the longest running study, conducted over 21 weeks, saw no improvement in cognitive function, and little improvement in psychosocial function compared to psychotherapy [49]. This intervention was not computer-assisted and provided participants with only one CT session per week, which may have been less than optimal. There was also little evidence of cognitive transfer effects, as improvements are observed in trained cognitive domains only. Improvements in cognitive function, depression, and quality of life at follow-up suggest that improvements may take time to be observed, particularly in terms of cognitive improvements. Future studies should be conducted to determine if these improvements are potentially due to neuroplastic changes, or to participants utilising strategies that they learnt in training.

There is some evidence that CT may improve eating disorder severity. However, this was only observed in one study and not reflected in Body Mass Index (BMI) or other quality of life, mood, or eating disorder measures [50]. This may be due to participants concomitantly receiving intensive therapies for their eating disorder which are superior to CT for an eating disordered cohort. Additional studies must be conducted to determine whether CT may provide a beneficial addition to standard care. Future studies may benefit from utilising a less severe cohort of participants with disordered eating and comparing CT to less intensive or non-psychological therapies.

More than half of all studies that found no effect of CT were conducted with inpatients, who were likely to be experiencing more severe impairment, and therefore may have required larger doses of CT than the three to six weeks they had received. Inpatients and residential patients were also receiving extensive therapeutic care, involving psychotherapy, cognitive behavioural therapy, dialectical behavioural therapy, social skills training, medical treatment, music and exercise therapies, and group interventions [43,44,50]. One study that included a second intervention group receiving psychoeducation saw similar improvements in CT and psychoeducation compared to controls [49]. Previous studies in anorexia nervosa compared CT to art therapy and found similar improvements in eating disorder psychopathology, BMI, and cognitive function [54]. These findings suggest that participants may benefit from activities that encourage self-reflection, learning, and social engagement, regardless of the specific behaviour or cognitive function targeted.

Two studies that saw improvements in depressive symptoms also saw improvements in attention or memory, suggesting that improvements in depressive symptomatology may be related to improvements in cognitive function. Cognitive training can induce plasticity and increased neural activity in the prefrontal cortex, a region critical for working memory and executive functioning [55]. Rumination and perseverative, self-focused thoughts have been associated with enhanced activation of the dorsomedial prefrontal cortex and limbic regions [56] and hypoactivation of the dorsolateral prefrontal cortex [57]. Strengthening cognitive control over limited working memory content may increase working memory capacity and reduce rumination and depression. Attention training has also been used to reduce depression, and may provide a superior target for CT, although past results for this form of training are inconsistent [19]. As increased rumination is linked to more severe and sustained depression and poor response to pharmaceutical or cognitive behavioural therapies, CT may be a beneficial addition to standard therapies for treatment-resistant depression, or a standalone treatment for mild to moderate depression. However, further investigation using consistent CT approaches and measures would be required to substantiate this hypothesis.

Studies investigating SUD showed mixed results. A study in methamphetamine use saw improvements in working memory along with improvements in decision making and risk taking [44]. One study in alcohol use disorder found improvements in working memory and cognitive flexibility, but included no measure of addictive symptoms to allow investigation of the relationships between cognitive and addiction [45]. Further studies need to include both measures of cognitive function and addiction symptoms to infer a relationship between CT and improvements in SUD. Studies were also short, at only four to five weeks in duration. Previous studies investigating computerised CT in SUD that identified improvements in cognitive function and abstinence were conducted for eight weeks [58,59], suggesting a longer duration may be required.

The application of CT for bipolar affective disorder demonstrated potential psychosocial benefits. However, studies did not include a validated measure of specific bipolar symptomatology such as mania or depression. It is therefore unknown how cognitive remediation may benefit this group in terms of disorder-specific symptoms and how cognitive function may relate to bipolar symptomatology. Computerised Interactive Remediation of Cognition and Thinking Skills (CIRCuiTS) computer-assisted CRT showed the greatest benefits in cognitive improvements and psychosocial function [48]. However, this intervention would need to be compared to other therapies to find out its true benefit.

Only one eligible study in OCD was identified [27]. This study delivered therapist-led visual organisational strategies and was the only domain-specific training that reported improvements in a trained cognitive domain and in disease symptomatology (OCD symptoms). This finding may suggest that single-domain training could be beneficial, providing it targets the correct domain. There is also the possibility that any benefit seen subsequent to CT is due to it providing participants with a distraction. Distraction techniques have been found to improve emotion regulation and reduce depressive symptoms [60]. Previous research that found similar benefits from CT training and control groups including word puzzles [61] or other forms of therapy suggests that the act of being distracted may be the potent factor, rather than the cognitive training itself.

## 6. Strengths and Limitations

This study included only RCT studies without active control groups, which strengthened the reliability of findings. While active control groups are considered a gold standard for identifying true intervention effects, this is only true when the active control matches the intervention in engagement, action, and expectation without being exposed to the training aspects of the intervention [62]. Active control groups used in identified CT articles used a range of active control groups that did not adhere to this convention. Control groups included alternative cognitive training interventions, art therapy, computer games, and forms of talk therapy. Most did not match the intervention sufficiently, and many included control activities that may have also led to improvements in cognition or psychiatric symptoms which would have confounded results. The primary reason to include active controls is to avoid placebo effects. A previous meta-analysis of cognitive interventions employing active and passive control groups found no significant difference in performance, and no evidence of placebo effect [63].

There were a number of limitations in terms of study characteristics. Computerised training showed the greatest promise in terms of cognitive outcomes, although only one study that independently used (without therapist guidance) computerised CT intervention was identified, and computer-assisted training methods incorporated varying amounts of therapist input. Included studies showed no consistency in terms of CT type, delivery, cognitive domains targeted, or outcomes measured. For this reason, a meta-analysis of the findings would have included a high risk of unreliable and misleading findings. Studies also included a wide range of different analytic techniques, further complicating a meta-analysis. Small sample sizes mean that we cannot rule out biological or environmental variability as the cause for any change in cognition or function reported. More than one-third of the studies did not include a validated measure of disease symptomatology, and when included, no consistency was observed. There is also a great deal of inconsistency in the literature regarding CT and CRT. A white paper by Bowie and colleagues has defined CRT as requiring the inclusion of a number of specific elements such as a trained therapist, strategy coaching, and a focus on transfer of strategies to real world demands [4]. A number of papers have described their interventions as CRT while failing to incorporate a number of these aspects into their training intervention.

The small number of rigorous and consistent studies conducted within any psychiatric disorder outside schizophrenia provides insufficient evidence to determine the impact of CT in psychiatric disorders. Previous literature suggests that CT has no greater impact on cognition or psychiatric symptoms than psychotherapy, CBT, or word puzzles. A review of studies that have incorporated methodologically similar active control groups, in community members that are not receiving additional intensive treatment for their psychiatric disorder, may reveal whether or not CT is in fact superior or equal to alternative treatment methods.

## 7. Conclusions

Overall, no clear relationships were seen between CT dose or type and cognitive, neuropsychiatric, or behavioural outcomes. Nearly all included studies saw an improvement in at least one trained cognitive domain or psychiatric symptom measure, suggesting a benefit of CT, but there is little evidence of transfer effects to other domains. There was some evidence that CT may lead to improvements in disease symptomatology and function, but inconsistent findings cannot rule out an alternative cause to these improvements. Insufficient evidence was found to conclude a significant benefit of CT for individuals with a psychiatric disorder other than schizophrenia. Standardisation of CT format, delivery, and evaluation is required. More rigorous studies, in larger samples and of longer duration, are needed to determine the efficacy of CT.

## Figures and Tables

**Table 1:** Included article characteristics.

Author, Year	Study Characteristics			Training/Intervention Characteristics			Study Outcomes	
	Country	Sample N; Age (SD); Female	Diagnosis (Criteria)	Intervention	Format	Duration (Minutes) and Frequency	Training Tasks	Main Findings
Bowie, 2013 [38]	Canada	N = 33 outpatients I: n = 17; 49.2 (11.8); F = 11 C: n = 16; 42.2 (13.4); F = 12	Treatment-resistant MDD	Scientific Brain Training Pro Control—Wait list	Group, computerised	Length: 90 Sessions: 10 Weeks: 10	Exercises with adaptive difficulty and strategy coaching, targeting processing speed, attention, working memory, delayed memory, and EF.	↑Attention and processing speed (*p* = 0.012) ↑Verbal memory (*p* = 0.023) EF (ns) Social function (ns)
Choi, 2017 [39]	USA	N = 59 inpatients I: n = 21; 39.0 (12.7); F = 12. C1: n = 20; 41.7 (14.4); F = 12. C2: n = 18; 48.2 (10.8); F = 8.	MDD (DSM-IV)	Memory training for ECT (Mem-ECT) Control (C1)-TAU	Pen-and-paper, computerised	Length: 60–75 Sessions: 7 Weeks: 3	Memory strategies and skills.	Cognitive status (ns) Memory (ns) Subjective cognition (ns) Quality of life (ns) Depression (ns)
Klojčnik, 2023 [40]	Slovenia	N = 20 outpatients I: n = 10; 43.6 (11.5), F = 6 C: n = 10; 47.0 (9.2); F = 6	Depression (ICD 10)	Cogniplus Control-TAU	Therapist-led, computerised	Length: 40–50 Sessions: 12 Weeks: 10	Tasks with adaptive difficulty, training attention, and EF.	↑EF (*p* < 0.000 to 0.021) ↑Attentional performance (*p* < 0.000 to *p* = 0.007) ↓Depression (*p* = 0.006) ↑Self-reported cognition subscales of shifting, emotional control, initiate: (*p* < 0.000) Self-reported cognition subscales of inhibition (ns) working memory (ns) Planning (ns)
Listunova, 2020 [41]	Germany	N = 57 community members aged 18–60. I (GT): n = 18; 45.3 (15.1); F = 14 I (IT): n = 20; 45.9 (11.3); F = 15 C: n = 19; 44.9 (10.3); F = 13	MDD (DSM-IV)	Cogniplus GT (generalised training): trained all cognitive domains IT (individualised training): trained three worst domains Control—no care	Computerised + therapist-led sessions.	Length: 60 Sessions: 12–15 Weeks: 5	Tasks with adaptive difficulty, training attention, alertness, working memory, inhibition, planning + weekly transfer sessions with psychoeducation on cognition and strategy training.	No difference between GT and IT. ↑Psychosocial function (*p* = 0.014) ↑Attention (*p* = 0.014) Information processing (ns) EF (ns) Learning and memory (ns) Depression (ns)
Neshat-Doost, 2013 [42]	Iran	N = 23 adolescent students I: n = 12; 15.3 (1.7); F = 6 C: n = 11; 15.5 (2.1); F = 5	Depression (MFQ)	Memory specificity training (MEST) Control—no care	Group, therapist-led	Length: 80 Sessions: 5 Weeks: 5	Memory education, training, and practice.	↑Retrieval of specific autobiographical memories (*p* < 0.01). ↓Depression at 2-months follow-up (*p* = 0.04). Autobiographical memory specificity predicted follow-up depression (*p* < 0.01).
Trapp, 2016 [43]	Germany	N = 46 inpatients I: n = 23; 34.26 (11.60); F = 14 C: n = 23; 36.87 (12.14); F = 17	Depression (DSM-IV and ICD-10)	X-Cog Control-TAU	Group, therapist-led, computerised training.	Length: 60 Sessions: 12 Weeks: 4	Game-like tasks with increasing difficulty and strategy development, targeting visuomotor, memory, EF, attention.	↑Working memory (*p* = 0.030) ↑Memory (*p* = 0.006) ↑Cognitive flexibility (*p* = 0.019) Depression (ns)
Brooks, 2017 [44]	South Africa	N = 35 inpatients I: n = 20; 29.83 (7.32) Control: n = 15; 28.11 (6.01)	Methamphetamine use disorder	Curb Your Addiction Control—TAU	Group, therapist-led, computerised.	Length: 30 Sessions: 20 Weeks: 4	N-back task, training working memory with adaptive difficulty.	Mood (ns) Craving (ns) Self-control (ns) Impulsivity (ns) Self-regulation (ns) Anxiety (ns) Depression (ns) Executive function (ns)
Gamito, 2021 [45]	Portugal	N = 36 adult residential patients, F = 6. I: n = 19; 40.0, (9.2) C: n = 17 51.0 (12.3)	Alcohol use disorder	Systemic Lisbon Battery (SLB) Control—TAU	Individual, therapist-led sessions.	Length: 30–40 Sessions: 10 Weeks: 5	Virtual reality (VR)-based cognitive intervention targeting memory, attention, and EF in real-life scenarios	↑Attention (*p* = 0.03 to *p* = 0.02). ↑Cognitive flexibility (*p* = 0.001) Visual perception (ns) Memory (ns)
Zhu, 2018 [46]	China	N = 40 Male inpatients I: n = 20; 32.7 (5.3) C: n = 20; 35.1 (8.0)	Methamphetamine use disorder (DSM-V)	Mobile-based computerised cognitive addiction therapy App Control—TAU	Independent, computerised training.	Length: 60 Sessions: 20 Weeks: 4	Working memory and attentional bias training plus relaxation.	↑Verbal learning and memory (*p* < 0.001) ↑Spatial working memory (*p* < 0.001) ↑Decision making (*p* < 0.001) ↑Risk taking (*p* < 0.001) Problem-solving (ns) Attentional bias (ns)
Demant, 2015 [47]	Demark	N = 40 outpatients aged 18–50. I: n = 18; 33.9 (6.8); F = 12 C: n = 22; 34.0(7.9); F = 13	Partially of fully remitted Bipolar (ICD-10)	RehaCom Control—TAU	Group, therapist education and computerised training	Length: 120 Sessions: 12 Weeks: 12	Computer tasks plus psychoeducation strategy training targeting memory, attention, and EF.	↑Verbal fluency (*p* = 0.005) and quality of life (*p* = 0.048) at 12 weeks follow-up. Verbal memory (ns) Attention (ns) EF (ns) Psychosocial function (ns) Psychomotor speed (ns)
Strawbridge, 2020 [48]	England	N = 60 community members aged 18–65. I: n = 29; median (IQR) = 43 (34, 52.5); F = 21 Control: n = 31; median (IQR) 42.5 (31.8, 52.2); F = 20	Bipolar	CIRCuiTS Control—TAU	Therapist-led, computerised training.	Sessions: ~36 Weeks: 12 Minimum 20 h of training	Drill and practice tasks plus strategy-based approaches targeting attention, memory, and executive function.	Week 13: ↑Working memory (*p* = 0.024) ↑IQ (*p* = 0.015) ↑Executive function (*p* = 0.027) ↑Functional capacity (*p* = 0.015) ↑Psychosocial functioning (*p* = 0.004) ↑Goal attainment (*p* < 0.001) Week 25: ↑Processing speed and attention (*p* < 0.05) ↑Working memory (*p* = 0.001) ↑Verbal memory (*p* = 0.007) ↑IQ (*p* = 0.001) ↑Verbal fluency (*p* = 0.004) ↑Executive function (*p* = 0.003) ↑Global cognition (*p* = 0.001) ↑Psychosocial functioning (*p* = 0.002) ↑Goal attainment (*p* = 0.001)
Torrent, 2013 [49]	Spain	N = 239 outpatients aged 18–55. I1: n = 77; 40.6 (9.1) I2: n = 82; mean age = 39.3 (8.9) C: n = 80, 40.5 (8.7)	Bipolar in remission (DSM-IV)	Functional remediation Control—TAU I1: Functional remediation I2: Psychoeducation	Group, pen-and-paper	Length: 90 Sessions: 21 Weeks: 21	Training and strategy coaching targeting attention, memory, problem-solving, multitasking, and organisation.	↑Functioning compared to TAU (*p* = 0.002) but not compared to psychoeducation (*p* = 0.056). Improvements were seen in ‘interpersonal’ and ‘occupational’ domains of function. Executive function (ns) Set shifting (ns) Planning (ns) Inhibition (ns) Verbal fluency (ns) Memory (ns) Attention (ns)
Dingemans, 2014 [50]	Netherlands	N = 82 female inpatients aged 17–53. I: n = 41; 26 (22) C: n = 41; 23 (21)	Anorexia Nervosa (DSM-IV)	Manualised cognitive remediation therapy for AN Control—TAU	Therapist-led, individual, pen-and-paper sessions.	Length: 45 Sessions: 10 Weeks: 6	Exercises, pychoeducation, strategy coaching, and reflection target cognitive flexibility and information processing.	↓EDQoL at 6 weeks (*p* < 0.05) ↓EDEQ at 6 months (*p* < 0.05) BMI (ns) Quality of life (ns) Depression (ns) Anxiety (ns) Self-esteem (ns) Perfectionism (ns) Motivation to change (ns) Neuropsychological functioning (ns)
Sproch, 2019 [51]	USA	N = 275 inpatients I: n = 135; 23.9 (12.8); F = 121 C: n = 140; 22.2 (12.8); F = 129	Anorexia Nervosa (DSM-IV)	Manualised cognitive remediation therapy for AN Control—TAU	Therapist-led, group, pen-and-paper sessions.	Length: 60 Sessions: 5 Weeks: 3	Exercises, pychoeducation, strategy coaching, and reflection targeting cognitive flexibility.	Executive function (ns) Cognitive flexibility (ns) Age by group analysis provided evidence that adults may benefit more than children from CRT on cognitive flexibility.
Park, 2006 [27]		N = 30 outpatients I: n = 15; 30.5 (10.4); F = 5 C: n = 15; 28.1 (6.8); F = 4	OCD (DSM-IV)	Revised block design subtest of K-WAIS.	Therapist-led	Length: 60 Session: 9 Weeks: 5	Training of organisational strategies.	↑Visuospatial construction ability (*p* < 0.05) ↑Visuospatial memory (*p* < 0.01 to *p* < 0.05) Verbal learning and memory (ns) Better performance for control condition on trial B recall. ↓Obsessions (F = 13.7, *p* < 0.01) ↓Compulsions (F = 6.8, *p* < 0.05) ↓OCD symptom severity (F = 19.8, *p* < 0.001) Depression (ns) Anxiety (ns)

Abbreviations: ↑, increase in score or symptom; ↓, decrease in score or symptom; AN, anorexia nervosa; BMI, Body Mass Index; C, control; CRT, cognitive remediation therapy; DSM, Diagnostic and Statistical Manual of Mental Disorders; ECT, electroconvulsive therapy; EDEQ, Eating Disorder Examination Questionnaire; EDQol, Eating Disorder Quality of Life scale; EF, executive function; I, intervention; ICD, International Classification of Diseases; IQ, Intelligence Quotient; IQR, interquartile range; MDD, major depressive disorder; MFQ, Mood and Feelings Questionnaire; ns, not significant; OCD, obsessive compulsive disorder; TAU, treatment as usual.

## Supplementary Materials

The following supporting information can be downloaded at: https://www.mdpi.com/article/10.3390/jcm13154348/s1, Figure S1: Risk of bias summary.
