# Supplementary material for: A Systematic Review and Narrative Synthesis of Cognitive Training in the Treatment of Mental Illness and Substance Use Disorder
Source: J Clin Med. 2024 Jul 25;13(15):4348. doi: 10.3390/jcm13154348 (PMC11312778; doi:10.3390/jcm13154348)
Supplement: Supplementary file 1 [file jcm-13-04348-s001.zip › jcm-3055559-supplementary.pdf]

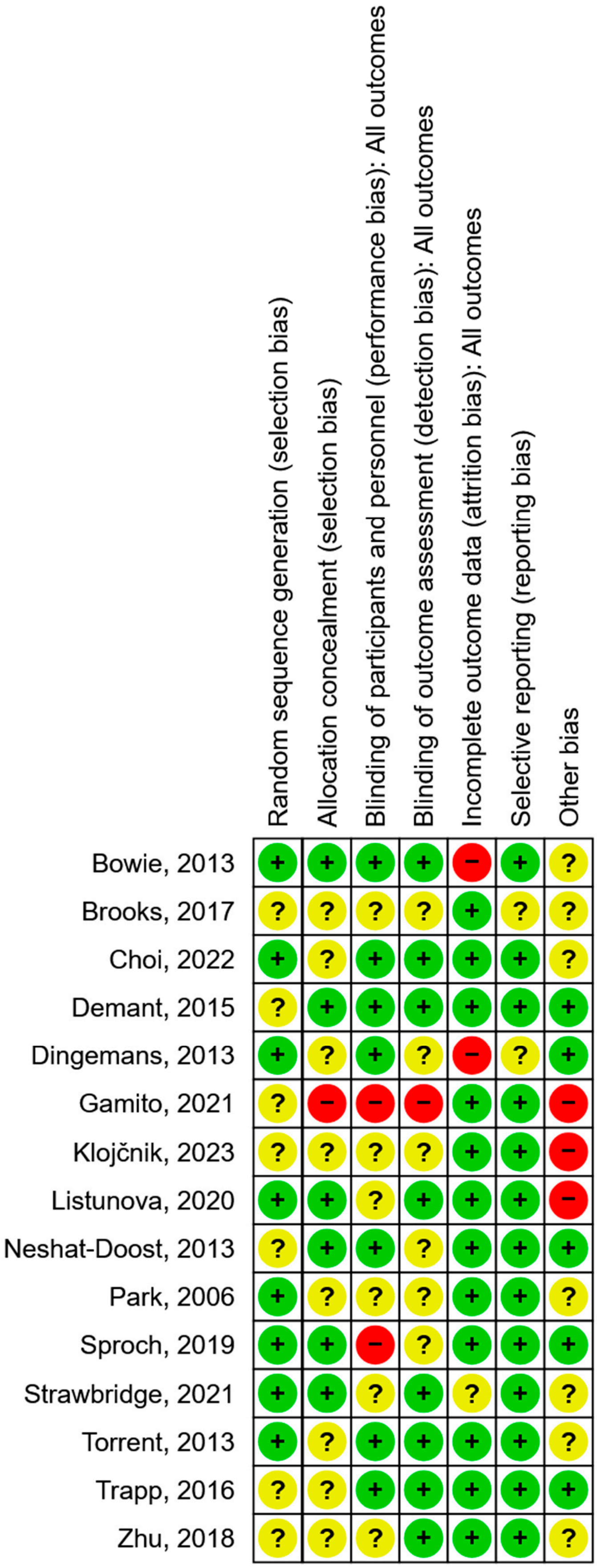

Figure S1: Risk of bias summary [27,38,44,39,47,50,45,40,41,42,43,46,48,49,51].
